# A Cool Proposition

**Published:** 1912-07

**Authors:** 


A Cool Proposition.—The independent medical journals have again been marked for slaughter, and the owners are asked how they will take it; how they would prefer to be killed.

Dr. I. N. McCormack, of Bowling Green, Ky., who, it will be remembered, had his sins forgiven by the Governor when he was indicted for a violation of the sanitary ordinances of his town, issues a document and sends a copy of it to the independent medical journals, which, for cool impertinence, excels anything I have recently seen.

Dr. Murphy, President A. M. A., recommended that the A. M.

A. issue a dollar journal, which, in addition to other things, should "carry personal news, medical economics, courses of instruction, legal decisions on medical subjects, write-ups on climate, States, cities, hospitals and men; in other words, a breezy, gossipy, personal, helpful magazine, containing * * * the most modern medical facts." In other words, it should follow the style and arrangement of the independent journals—of which the "Red Back," for instance, is a type and representative.

Dr. McCormack is chairman of a committee to report upon the feasibility of Dr. Murphy's suggestion, and to that end he wants to know of us, and other sinners:

"Whether the medical profession shall be forced to supplant American medical journals as at present conducted, many of them knowingly carrying advertisements and reading notices of medical and other frauds, and, therefore, owing their first allegiance to such patrons rather than to the medical profession, with journals owned or controlled by reputable physicians or organizations."

He assumes that issuing such a journal would "supplant" our kind, and proposes that the supplanter shall be "owned or controlled by *reputable physicians* or organizations" [instead of by such as Steadman, Taylor, Lewis, Sajous, Winslow Anderson, Register, Coe *et al.*, to say nothing of the writer].

Well, if that isn't cheek! Especially seeing that the great A. M. A. journal and some of its satellites are now carrying the very kind of advertisements that it is alleged make the independent journals "disreputable." But it excites one's risibles to contemplate the Octopus trying to issue an imitation of the independent type which shall be "a breezy, gossipy, personal, helpful magazine." Why, I should as soon expect to see an owl laugh or a jolly funeral procession, for the Octopus is as solemn as the one and as gruesome as the other.

We have not sent in our opinion yet. The Chicago oligarchy has tried every other method of killing off the independents, let them now try this plan. It is kind of them to ask us how we like it. Send along your "reputable physicians" to "supplant" the "Red Back."

---

THE STATE MEDICAL ASSOCIATION, through its Committee on Legislation, will ask for a "State Institution for the Care of Inebriates." Why not stop the sale of liquor, and we wouldn't have any inebriates?

